# Deep peroneal nerve palsy with isolated lateral compartment syndrome secondary to peroneus longus tear: a report of two cases and a review of the literature

**DOI:** 10.1007/s10195-015-0373-8

**Published:** 2015-09-11

**Authors:** Kunihiko Hiramatsu, Yasukazu Yonetani, Kazutaka Kinugasa, Norimasa Nakamura, Koji Yamamoto, Hideki Yoshikawa, Masayuki Hamada

**Affiliations:** Department of Orthopaedic Surgery, Yao Municipal Hospital, 1-3-1, Ryugecho, Yao, Osaka Japan; Department of Orthopaedic Surgery, Hoshigaoka Medical Center, 4-8-1, Hoshigaoka, Hirakata, Osaka Japan; Institute for Medical Science in Sports, Osaka Health Science University, Osaka, Japan; Center for Advanced Medical Engineering and Informatics, Osaka University, Suita, Osaka Japan; Department of Orthopaedic Surgery, Toyonaka Municipal Hospital, 4-14-1, Shibahara, Toyonaka, Osaka Japan; Department of Orthopaedic Surgery, Osaka University Graduate School of Medicine, 2-2, Yamada-oka, Suita, Osaka 565-0871 Japan

**Keywords:** Lateral compartment syndrome, Deep peroneal nerve palsy, Peroneus longus tear

## Abstract

Drop foot is typically caused by neurologic disease such as lumbar disc herniation, but we report two rare cases of deep peroneal nerve palsy with isolated lateral compartment syndrome secondary to peroneus longus tears. Both patients developed mild pain in the lower legs while playing sport, and were aware of drop foot. As compartment pressures were elevated, fasciotomy was performed immediately, and the tendon of the peroneus longus was completely detached from its proximal origin. The patients were able to return their original sports after 3 months, and clinical examination revealed no hypesthesia or muscle weakness in the deep peroneal nerve area at the time of last follow-up. The common peroneal nerve pierced the deep fascia and lay over the fibular neck, which formed the floor of a short tunnel (the so-called fibular tunnel), then passed the lateral compartment just behind the peroneus longus. The characteristic anatomical situation between the fibular tunnel and peroneus longus might have caused deep peroneal nerve palsy in these two cases after hematoma adjacent to the fibular tunnel increased lateral compartment pressure.

## Introduction

Compartment syndrome of the lower extremity is a rare event and can occur with trauma or occasionally with a sports injury. The diagnosis needs to be established early in its course to avoid disabling sequelae, such as neurologic disorders. The most frequent location of compartment syndrome in the lower extremity is the anterior compartment. Looking at the literature, lateral compartment syndrome of the lower leg is quite rare. Lateral compartment syndrome occurs due to inversion ankle injuries [1, 2], exertion [3, 4], horseback riding [5, 6], a prolonged lithotomy position in general surgical, urologic, and gynecologic procedures [7], peroneus longus muscle tears or avulsion [8–12].

Early diagnosis and treatment of lateral compartment syndrome secondary to peroneus longus tear is difficult due to the lack of characteristic clinical symptoms [3, 13]. To the best of our knowledge, deep peroneal nerve palsy with lateral compartment syndrome secondary to complete avulsion of the proximal origin of the peroneus longus has not been reported. Two rare cases of deep peroneal nerve palsy with isolated lateral compartment syndrome secondary to peroneus longus tear are reported herein.

## Case reports

### Case 1

**Fig. 1:**
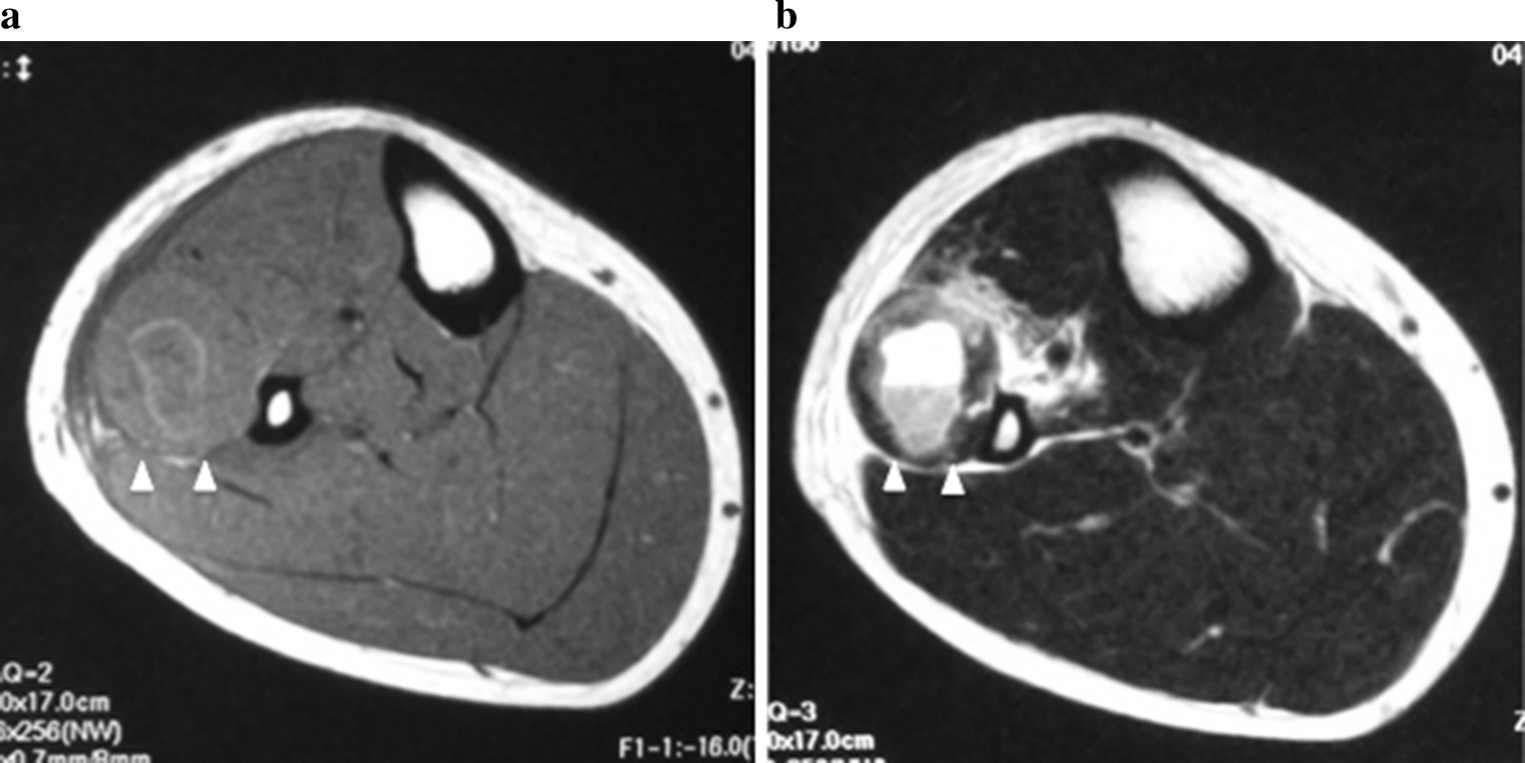
MRI of the right knee (case 1). Axial T1-weighted fast spin echo (**a**), and T2-weighted fast spin echo (**b**). *Arrowheads* indicate hematoma in the peroneus longus muscle, which shows a fluid–fluid level

A 21-year-old man who had previously experienced no pain in the legs, no muscle weakness, and no other disorders, developed mild pain in the right lower leg while playing baseball, but he was able to continue to playing. Three days later, he became aware of drop foot of the right leg, but did not seek medical care because he could tolerate the pain. Two days later, he presented to the orthopedic department complaining of persistent drop foot of the right leg. The initial clinical examination revealed mild swelling of the anterior and lateral right lower leg, with focal prominence over the lateral muscle compartment, as well as pain and tenderness. No pain was evident with passive plantar flexion of the ankle, and plantar flexion of the digits was elicited. Manual muscle testing revealed 0/5 muscle strength of the anterior muscle group (tibialis anterior and extensor hallucis longus), 5/5 muscle strength of the posterior muscle group, and 0/5 muscle strength of the peroneus muscle. The posterior tibial and dorsalis pedis artery pulses were both palpable. There was decreased sensation in the deep peroneal nerve area, but sensation was normal in the superficial peroneal nerve area. Lumbar diseases such as disc herniation were excluded because patella tendon and Achilles tendon reflexes were normal, and the straight leg raising test yielded negative results. Magnetic resonance imaging (MRI) of the right lower leg was performed because of swelling of the anterior and lateral lower right leg. MRI demonstrated hypointensity on T1-weighted imaging and hyperintensity on T2-weighted imaging that appeared to represent a cystic lesion with fluid–fluid level findings in the lateral compartment (Fig. 1a, b), and pressures in the anterior and lateral compartments were 42 and 120 mmHg, respectively. Based on these clinical findings, fasciotomy was performed based on a definitive diagnosis of anterior and lateral compartment syndromes.

The proximal lower leg was exposed through a longitudinal incision above the lateral compartment. Hematoma within the lateral compartment was evacuated. The tendon of the peroneus longus was found to be completely detached from its proximal origin. Although the hematoma was evacuated, lateral compartment pressure remained elevated (120 mmHg). The distal lower leg was exposed through an incision above the lateral compartment. The peroneus muscle was ischemic and swollen, but not necrotic. The skin was closed, because the skin was not tense. Reduced lateral compartment pressure was confirmed, and the operation was finished. The day after the operation, the patient complained of right lower leg pain. The wound was opened because lateral compartment pressure was again increased (120 mmHg). After the wound was opened, the patient noted pain relief.

Fourteen days later, he was taken back to the operating room for delayed primary closure. At the time of primary closure, tibialis anterior strength had recovered to 3/5, and extensor hallucis longus and peroneus strengths were 1/5. The patient was discharged 18 days after fasciotomy, requiring an ankle–foot orthosis for ambulation. Three months after fasciotomy, he was able to return to play baseball with almost complete recovery of muscle strength in the tibialis anterior (5/5) and extensor hallucis longus/peroneus (4/5). Clinical examination after 2 years revealed no hypesthesia and no muscle weakness in the territory of the deep peroneal nerve.

### Case 2

A 16-year-old boy with no history of pain in the leg, muscle weakness, or other disorders developed pain in the right lower leg after playing soccer. Sixteen days later, he presented to the orthopedic department complaining of swelling, pain, and numbness in the right leg. The initial clinical examination revealed swelling of the right lower leg, and manual muscle testing showed 4/5 muscle strength of the anterior muscle group (tibialis anterior and extensor hallucis longus), 5/5 muscle strength of the posterior muscle group, and 3/5 muscle strength of the peroneus muscle. Sensation was decreased in the deep peroneal nerve area, but normal in the superficial peroneal nerve area. Anterior compartment pressure was 42 mmHg, but that of the lateral compartment was 100 mmHg. The results of these clinical examinations led to the definitive diagnosis of anterior and lateral compartment syndromes, and fasciotomy was immediately performed.

**Fig. 2:**
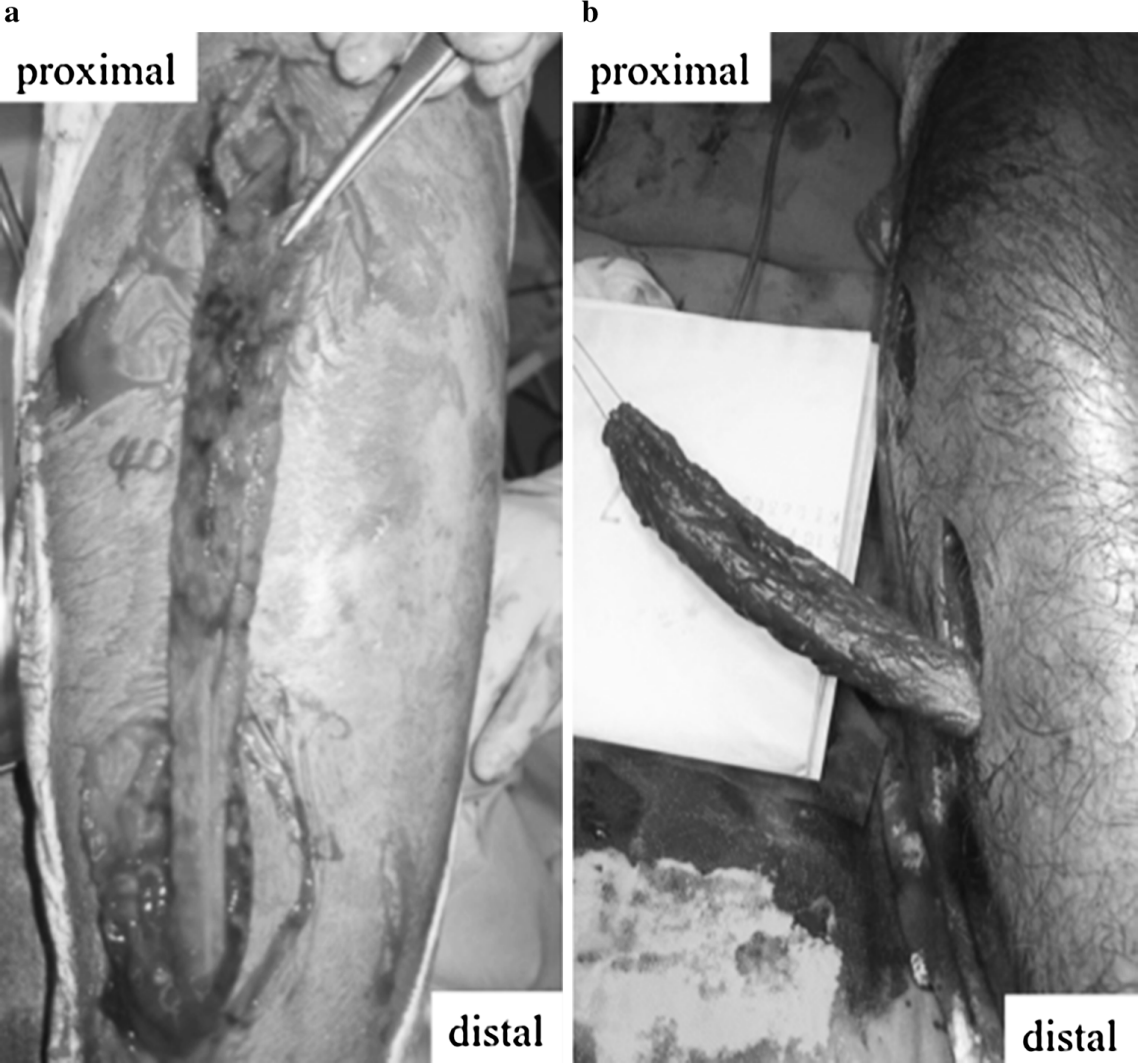
Intraoperative photograph of the leg. The peroneus longus is completely detached from its proximal origin and retracted distally out of the lateral compartment. **a** Case 1, **b** case 2

During fasciotomy through a longitudinal incision in the lateral side of the leg, the tendon of the peroneus longus was found to be completely detached from its proximal origin (Fig. 2). After confirmation of decreased lateral compartment pressure, the operation was finished. Nine days later, wound closure was performed without complications. One month after fasciotomy, muscle testing revealed full strength had been regained, and he could fully return to play soccer.

## Discussion

This is the first case report of deep peroneal nerve palsy with isolated lateral compartment syndrome secondary to peroneus longus tears. In both cases, it was difficult to diagnose because of the few and complex symptoms, such as drop foot, which often occur in lumbar disc herniation.

Although the most frequent presentation of compartment syndrome of the lower extremity involves the anterior compartment, lateral compartment syndrome of the leg is rare. In addition, as injury to the peroneal muscle–tendon unit tends to occur more distally, reports of acute rupture of the peroneus longus muscle from its proximal origin are very rare [14]. To the best of our knowledge, only four cases of isolated lateral compartment syndrome secondary to peroneus longus tear have been described [9–12]. In those previous reports, the pathological processes causing the peroneus longus to tear from its proximal origin were initiated by overuse of muscles, but the situations of injury remained unclear [10, 11]. As in previous cases, the present two cases did not show the injury situations clearly. From these perspectives, peroneus muscle tear should be included in the differential diagnosis for patients who play sports intensely and develop lateral lower leg pain.

**Fig. 3:**
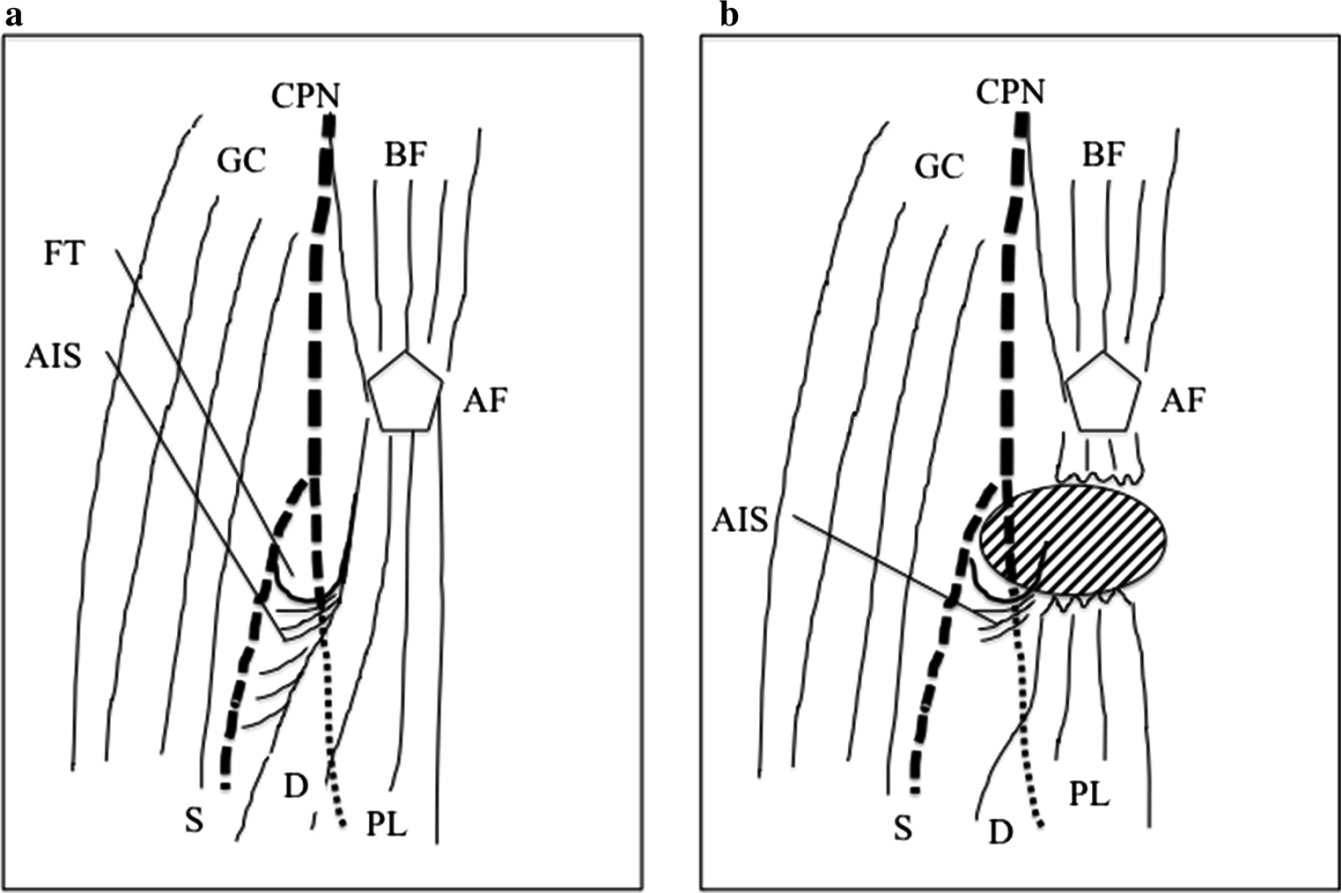
Schematic diagrams of the normal anatomy around the proximal end of the peroneus longus and peroneal nerve (**a**). Lateral compartment syndrome may result from a peroneus longus tear leading to peroneal nerve palsy (**b**). The common peroneal nerve (CPN) pierces the deep fascia and lies over the fibular neck, which forms the floor of the short ‘fibular tunnel’ (FT), and passes the lateral compartment just behind the peroneus longus. Idiopathic deep peroneal nerve entrapment can occur at the level of the fibular tunnel behind the peroneus longus, because hematoma beside the fibular tunnel increases lateral compartment pressure. *CPN* common peroneal nerve, *S* superficial peroneal nerve, *D* deep peroneal nerve, *BF* biceps femoris muscle, *AF* apex of the fibula, *FT* fibular tunnel, *AIS* anterior intermuscular septum, *PL* peroneus longus, *GC* gastrocnemius. The *oval* filled by *oblique lines* represents hematoma

The present two cases had unusual clinical findings for the following three pathognomonic symptoms. First, pain with passive stretching, which is one of the typical clinical signs of compartment syndrome, was absent. However, Lee et al. reported that a lack of pain with passive stretching might be due to rupture of the peroneus longus muscle [10]. Second, neuropathy was the main clinical manifestation in both of our two cases. Generally, when a young patient presents with deep peroneal nerve disorder, such as drop foot, lumbar disc herniation is usually suspected. Third, independent deep peroneal nerve palsy was present in both cases, although no case with independent deep peroneal nerve palsy has previously been described among the reports of isolated lateral compartment syndrome secondary to peroneus longus tear. Previous reports and the features of the course of the peroneal nerve usually lead to the conclusion that the neurological symptoms caused by isolated lateral leg compartment syndrome might not result in a deep peroneal nerve lesion, but rather in a superficial peroneal nerve lesion. However, a precise anatomical study showed that the common peroneal nerve pierces the deep fascia and lies over the fibular neck, which forms the floor of a short tunnel (the so-called fibular tunnel), and passes the lateral compartment just behind the peroneus longus [15]. At this fibular tunnel, the common peroneal nerve divides into the deep and superficial peroneal nerves. From these anatomical findings, Ryan et al. showed that idiopathic nerve entrapment could occur at the level of the fibular tunnel behind the peroneus longus [16]. Therefore, the characteristic anatomical situation between the fibular tunnel and peroneus longus might have caused the deep peroneal nerve palsy in our two cases when hematoma adjacent to the fibular tunnel increased lateral compartment pressure (Fig. 3).

In conclusion, we have reported two rare cases of deep peroneal nerve palsy with isolated lateral compartment syndrome secondary to peroneus longus tear. In both cases, diagnosis was difficult due to the few and complex symptoms, such as drop foot, which often occurs with lumbar disc herniation. Although rare, isolated lateral compartment syndrome secondary to peroneus longus tear should be considered in patients who play sports intensely and develop leg pain with peroneal nerve palsy.
